# Rapid and Impressive Response to a Combined Treatment with Single-Dose Tocilizumab and NIV in a Patient with COVID-19 Pneumonia/ARDS

**DOI:** 10.3390/medicina56080377

**Published:** 2020-07-27

**Authors:** Marco Cascella, Immacolata Mauro, Elvio De Blasio, Anna Crispo, Alfredo Del Gaudio, Sabrina Bimonte, Arturo Cuomo, Paolo Antonio Ascierto

**Affiliations:** 1 Division of Anesthesia and Pain Medicine, Istituto Nazionale Tumori-IRCCS-“Fondazione G. Pascale”, 80131 Naples, Italy; m.cascella@istitutotumori.na.it (M.C.); s.bimonte@istitutotumori.na.it (S.B.); a.cuomo@istitutotumori.na.it (A.C.); 2 Pneumology Unit, Ospedale Mauro Scarlato, 84018 Scafati (SA), Italy; i.mauro@aslsalerno.it; 3 Multidisciplinary Emergency Unit for COVID-19 Campania, 80100 Naples, Italy; elvio.deblasio@gmail.com; 4 Epidemiology and Biostatistics Unit, Istituto Nazionale Tumori-IRCCS-“Fondazione G. Pascale”, 80131 Naples, Italy; 5 DSC Anestesia e Rianimazione 2, IRCCS Casa Sollievo Della Sofferenza, 71013 San Giovanni Rotondo (FG), Italy; freddydelgaudio@libero.it; 6 Melanoma, Cancer Immunotherapy and Development Therapeutics Unit, Istituto Nazionale Tumori-IRCCS-“Fondazione G. Pascale”, 80131 Naples, Italy; p.ascierto@istitutotumori.na.it

**Keywords:** case report, cytokines, immunotherapy, inflammation, COVID-19, acute respiratory distress syndrome (ARDS)

## Abstract

Treatment of acute respiratory distress syndrome (ARDS) due to COVID-19 pneumonia (CARDS) represents a clinical challenge, requiring often invasive mechanical ventilation (IMV). Since the pathogenesis of CARDS it probably involves a direct viral attack to pulmonary and endothelium cells, and immune-mediated inflammation with dysfunctional coagulation, it was suggested to interfere with interleukin-6 (IL-6) activity by using the IL-6 receptor monoclonal antibody tocilizumab (TCZ). We reported the case of a 54-year-old 100 kg male COVID-19 patient (BMI 29) with severe respiratory insufficiency featuring dyspnea and hypoxia (SpO_2_ 89% on room; PaO_2_ 53 mmHg). Despite treatment with antiviral and non-invasive ventilation (NIV), after 24 h there was a progressive worsening of clinical conditions with higher fever (40 °C), increased dyspnea, and hypoxia (PaO_2_/FiO_2_ or P/F ratio of 150). The patient was at the limit to be sedated and intubated for IMV. He was treated with tocilizumab (8 mg/Kg i.v., single shot 800 mg) and NIV in the prone positioning. After only 96 h, the clinical, laboratory, and imaging findings showed incredible improvement. There was an important gain in oxygenation (P/F 300), a decrease of C-reactive protein values, and a decrease of the fever. Both the neutrophil-to-lymphocyte ratio (NLR) and the derived NLR ratio dropped down to 44%. Chest imaging confirmed the favorable response. This case suggested that for CARDS management efforts are needed for reducing its underlying inflammatory processes. Through a multiprofessional approach, the combination of IL-6-targeting therapies with calibrated ventilatory strategies may represent a winning strategy for improving outcomes.

## 1. Introduction

Pneumonia can represent a serious clinical expression of COVID-19. Of note, approximately 42% of hospitalized patients affected by the symptomatic SARS-CoV-2 infection develop the acute respiratory distress syndrome (ARDS) [1]. ARDS is characterized by hypoxemic respiratory failure of different degrees with bilateral lung infiltrates. Its treatment represents a clinical challenge and often requires tracheal intubation and invasive mechanical ventilation (IMV). In particular, in COVID-19-associated ARDS (CARDS) over 50% mortality has been reported [1].

Despite that at the beginning of the pandemic, early invasive mechanical ventilation was indicated as the optimal strategy for CARDS management, in COVID-19 pneumonia the clinical picture of severe hypoxemia contrasted with the typical ARDS respiratory mechanics. In most cases of CARDS, indeed, mechanical properties of the respiratory system surprisingly showed good pulmonary compliance [2] whereas in classic ARDS the lungs exhibit a reduced ability to stretch and expand itself (i.e., reduced compliance). Evidently, CARDS represents a separate chapter of ARDS and common therapeutic strategies must necessarily be updated [2,3]. Clinical experience, for example, suggested that non-invasive ventilation (NIV) has a role of primary importance in the management of CARDS as it can improve oxygenation, limiting the work of the respiratory muscles and preventing the onset of the patient self-inflicted lung injury (P-SILI) [2]. In this setting, adding the prone position to NIV could improve oxygenation, decrease respiratory effort, reducing self-induced lung injury, and avoid the need of intubation and invasive ventilation, which could be particularly useful in the case of reduced availability of intensive care unit (ICU) beds [4,5,6]. Nevertheless, as in CARDS there is often a rapid and sudden clinical worsening, which mainly affects the respiratory performance in terms of gas exchange, NIV can have serious limitations and not infrequently is it necessary to quickly resort to invasive mechanical ventilation. Furthermore, dyspneic patients with greater respiratory drive and work of breathing could not tolerate the prone position.

In this context, the pathophysiology of the disease can clarify many doubts, offering the possibility of pharmacological strategies, which, in turn, allow treating lung damage through less aggressive approaches. Although the exact pathogenesis of this COVID-19 pneumonia is still unclear, a complex cascade involving a direct viral attack toward pulmonary and endothelium cells as well as immune-mediated inflammation with dysfunctional coagulation seems to play a pivotal role [7]. For instance, previous investigations conducted on the coronavirus-induced severe acute respiratory syndrome (SARS) and the Middle East respiratory syndrome (MERS), focused on the so-called cytokine storm, expressed as high release of proinflammatory cytokines such as interleukin-6 (IL-6), tumor necrosis factor α (TNF-α), IL-1β, IL-8, and IL-12 as well as interferon gamma inducible protein (IP10), macrophage inflammatory protein 1A (MIP1A), and monocyte chemo attractant protein 1 (MCP1) [8]. IL-6 is a pleiotropic proinflammatory multifunctional cytokine produced by several cell types and can modulate the B-lymphocytes and T regulatory lymphocytes function. As increased tissue and serum levels of IL-6 are involved in the pathogenesis of many inflammatory and autoimmune processes, including those expressing cytokine release syndrome (CRS) features, it was suggested to interfere with IL-6 activity for improving CARDS outcome [9]. For this purpose, preliminary data from clinical studies indicated that tocilizumab (TCZ) could be a winning strategy for reducing the COVID-19-associated inflammatory cascade and, in turn, the severity of the disease [10]. TCZ is a recombinant humanized monoclonal antibody of the IgG1 class, targeting the soluble IL-6 receptor (sIL-6R) and the membrane receptor (mIL-6R). It is prescribed for rheumatoid arthritis, juvenile arthritis, giant cell arthritis, Castleman’s syndrome as well as for reducing toxicity in immune checkpoint inhibitors-treated patients with steroid refractory [11], and in cytokine release syndrome due to chimeric antigen receptor T cell therapies [12]. By interfering with the excessive and aberrant Sars-Cov-2-induced host immune response, the effect of therapeutic strategies for COVID-19 management, including approaches of mechanical ventilation, can be probably strengthened.

We report a case of CARDS managed with TCZ and NIV performed in prone positioning (PP). This combined approach of immunomodulatory therapy with PP-NIV led to a rapid clinical improvement associated with an evident gain in the chest computerized tomography (CT) scans.

## 2. Presentation of Case Report

A male 54-year-old 100 kg Caucasian patient, BMI 29, was admitted to the hospital for dyspnea and tachypnea (>30 breaths/min), fever (higher than 38°C), malaise, and dry cough. The clinical evaluation showed hypoxia with a peripheral oxygen saturation (SpO_2_) of 89% in room air. The partial pressure of oxygen (PaO_2_) was 53 mmHg.

The patient had no pre-existing comorbidities. He was conscious and hemodynamically stable. Laboratory tests indicated a white blood cell count (WBC) 5.1 × 10^9^/L in peripheral blood with a percentage of lymphocytes of 17.5% (0.89 × 10^9^/L), and of neutrophils of 75.0% (3.83 × 10^9^/L). The neutrophil-to-lymphocyte ratio (NLR) was 4.30 and the derived NLR ratio (d-NLR; neutrophil count divided by the result of WBC count minus neutrophil count) was 3.01. Eosinophils were 0.034 × 10^9^/L. The elevated C-reactive protein (CRP; 193 mg/L), lactate dehydrogenase (LDH; 467 U/L), and ferritin values (937 μg/mL) were also found. The D-dimer was 0.92 μg/mL and prothrombin time 12.2 s. IL-6 concentration in plasma was 93 pg/mL.

After a short cycle (5 min) of oxygen therapy with a 40% oxygen Venturi mask, NIV was started due to the lack of response. The ventilation was set in continuous positive airway pressure (CPAP) through an oro-nasal mask with end-expiratory positive airway pressure (PEEP) of 14 cm H_2_O, and a fraction of inspired oxygen (FiO_2_) of 60%. In this stage the patient′s condition was not optimal to obtain collaboration for pronation.

The diagnosis of COVID-19 was confirmed by real-time reverse transcriptase polymerase chain reaction, and he started therapy with lopinavir/ritonavir 400/100 mg (two 200/50 mg) tablets twice daily, hydroxychloroquine 400 mg (two 200 mg) tablets twice a day for the first day, then 200 mg twice a day, azithromycin (500 mg per day), and enoxaparin 1 mg/kg twice daily.

**Figure 1:**
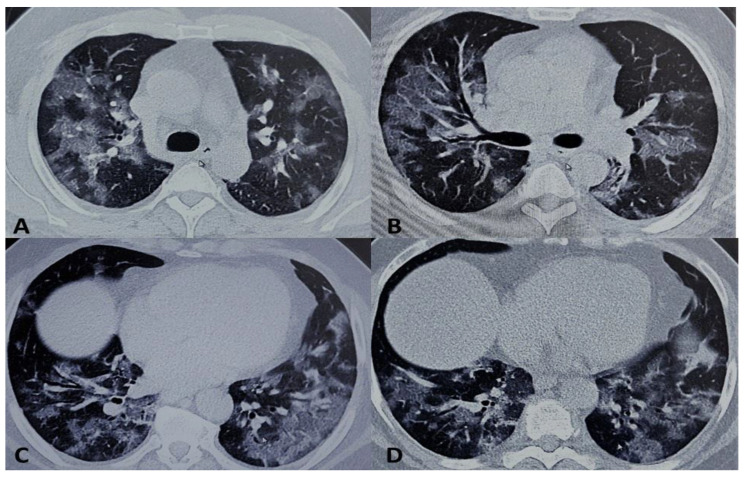
Computerized tomography (CT) scans tocilizumab pre-treatment. (**A**–**D**) Thin-slice (1 mm) axial unenhanced CT images demonstrated multiple patchy ground-glass opacity with a peripheral and subpleural distribution. Various reticular opacities are also detected within fields of ground glass (crazy-paving pattern). Carenal and prevascular lymphadenopathy is found.

Despite therapy, in the next 48 h the clinical picture showed no improvement, with a persistent fever (up to 40 °C), and a low PaO_2_/FiO_2_ ratio (150). CT scans confirmed the picture of a severe bilateral inflammatory process (Figure 1). Thus, the patient received tocilizumab (8 mg/kg i.v., 800 mg). After 2 h the fever disappeared and clinical condition progressively improved, allowing a trial of NIV in the prone position that was performed through three cycles of pronation per day. The mean period of each course of prone position was 90 min, with a good tolerance to the technique.

After 96 h from TCZ administration, there was a paramount clinical improvement. Dyspnea was resolved, and oxygen saturation was 96% with an important gain in oxygenation (PaO_2_/FiO_2_ ratio 300) and decrease of CRP (35 mg/L). There was a rise in lymphocyte percentage (24%; WBC of 3.6 × 10^9^/L with a count of lymphocytes of 0.86 × 10^9^/L). A decreased value was calculated for NLR (2.75) and d-NLR (1.92); both indices dropped up to 44%. There was 0.01 × 10^9^/L of eosinophiles. IL-6 concentration increased to 267 pg/mL.

**Figure 2:**
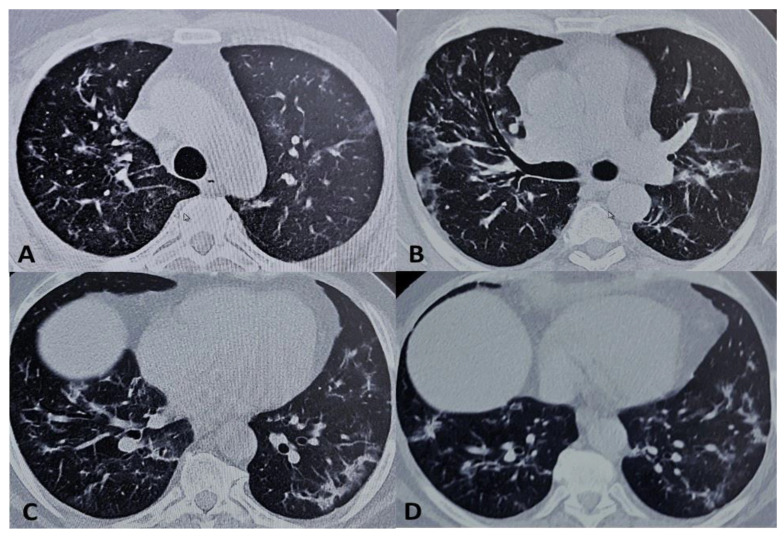
Computerized tomography (CT) scans after 96 h. Compared to pretreatment CT scans (Figure 1), (**A**–**D**) thin-slice (1 mm) axial unenhanced CT images showed an evident reduction (in terms of extension and density) of the bilateral lung interstitial infiltrates as well as of the extensive multiple patchy ground-glass opacities. Lymphadenopathy was also reduced.

The CT scans showed a significant improvement in the inflammatory pulmonary process. Although the pathological findings were not completely resolved, there was a rapid and evident reduction of the extensive multiple patchy ground-glass opacities demonstrated in the CT scans before TCZ treatment (Figure 2).

**Figure 3:**
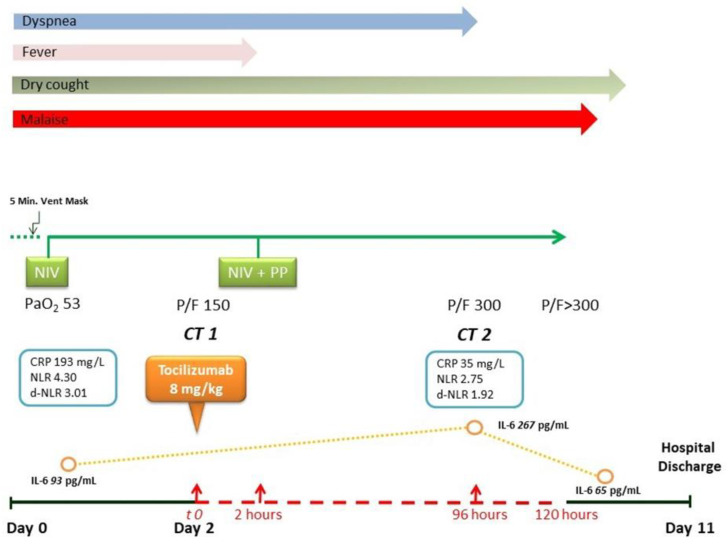
Timeline of the clinical course. Abbreviations: CT1: first computerized tomography; CT2: second computerized tomography; IL-6: interleukin-6; NIV: non-invasive ventilation; PP: prone positioning; CRP: C-reactive protein; P/F: PaO_2_ (partial pressure of oxygen)/fraction of inspired oxygen (FiO_2_) ratio; NLR: neutrophil-to-lymphocyte ratio; d-NLR: neutrophil-to-lymphocyte ratio (NLR).

Over the next 24 h, because the PaO_2_/FiO_2_ ratio was stable >300, we started the NIV weaning, by progressively reducing both PEEP and FiO_2_. Nine days after TCZ administration, the patient performed a SpO_2_ of 98% in room air, and he was discharged from the hospital completely recovered from COVID-19 symptoms (Figure 3).

## 3. Discussion

COVID-19 patients may have severe pictures of respiratory failure [13]. According to the Berlin Criteria for ARDS, a P/F value of 150 falls in the category of moderate ARDS. Although generally it corresponds to a mortality of 32% [14], in CARDS it is associated to a higher mortality [1].

While at the beginning of the COVID-19 crisis it was suggested to prefer the MIV adopting it after only a short challenge (1–2 h) of NIV—despite awareness that long time MIV treatments may lead to ventilator-induced lung injury (VILI) and higher risk of infection—a greater knowledge of the pathophysiology of the disease combined with the possibility of using drugs that may interfere with its pathogenic cascade, has drastically changed the therapeutic scenario and, consequently, outcomes. As a consequence, the premises for less aggressive lung ventilation approaches were created.

The rationale for NIV in ARDS is the application of the PEEP and PS. The former works by improving the functional residual capacity, opening collapsed alveoli, and by a redistribution of the perfusion into the lungs. The effect is an enhanced ventilation–perfusion ratio with a lowered intrapulmonary shunt and a better lung compliance. On the other hand, PS reduces muscle fatigue. These effects are enhanced by an early PP and by the possibility of performing pronation cycles for maintaining the advantage obtained [4,5,15]. Nevertheless, in a context of inflammatory syndrome with severe dyspnea associated with a poor general clinical picture, this therapeutic approach can be difficult to perform. The keystone may be the management of inflammatory underlying processes.

As previously found in SARS and MERS, elevated plasma levels of cytokines including IL-6 have been demonstrated also in CARDS patients. Moreover, these findings usually correlate to the severity of the clinical course [13]. IL-6 may have a key role in the inflammatory storm, which underlies the COVID-19 pathogenesis. Recently, Ciceri et al. [16] proposed the term of MicroCLOTS (microvascular COVID-19 lung vessels obstructive thromboinflammatory syndrome) for indicating the lung viral damage combined with the inflammatory reaction and the microvascular pulmonary thrombosis. Since in a scenario of multiple organ dysfunction syndrome these thromboinflammatory processes may extend beyond the boundaries of the lung, it is mandatory to identify treatments that, by acting on the pathogenic cascade, can enhance the efficacy of non-pharmacological approaches such as non-invasive ventilation and prone position, for instance through improved tissue oxygenation. Thus, although severe ARDS patients are probably not fitting candidates for NIV, this approach can be suggested also in CARDS featuring a lower PaO_2_/FiO_2_ ratio, especially if performed by adopting the prone position. Of note, despite that higher IL-6 levels were associated with the need for invasive ventilation due to a more severe disease [17], in preliminary data on CARDS patients treated with TCZ, favorable outcomes were observed in non-intubated patients [10,16,18]. Probably, interfering with the inflammatory storm when it is in its greatest acute phase could avoid the need to resort to aggressive therapies and timing of anti-cytokine therapy could be a key issue [19]. Specifically an early treatment with TCZ could turn off the inflammatory response and avoid the invasive treatments [20]. Interestingly, in our case the TCZ administration produced an important effect on the systematic inflammatory response expressed as decreased values of CRP, NLR, and d-NLR. Results from an Italian Phase II trial on TCZ for CARDS treatment (NCT04317092), and from the Phase 2/3, randomized, double-blind, placebo-controlled investigation on the use of the anti-IL-6R antibody sarilumab (NCT04315298), will certainly provide valuable information on efficacy and safety.

To date, although other reports have shown favorable outcomes and imaging improvement after TCZ administration [21,22], the peculiarity of our case was the rapid (96 h) and impressive positive results after only a single dose of TCZ.

## 4. Conclusions

Pending the results of ongoing clinical trials on IL-6-targeting therapies for COVID-19, our clinical practice suggests that due to the effects of the immune-modulatory agents and through a multiprofessional approach that exploits the skills of immunologist and intensivist/pulmonologist experts in mechanical ventilation, fewer and fewer patients are referred to IMV. In patients with severe CARDS, indeed, reducing lung functional impairment is a priority and often a decisive goal. On these bases, the ventilatory strategies of CARDS can be revised, resorting above all to the NIV and avoiding, when possible, IMV and possible ventilation sequelae such as P-SILI and VILI.

Although recently, other reports have shown a marked improvement in the clinical-radiological picture, the particularity of the case described is the speed with which the significant results were achieved. Probably, the possibility of combining a therapeutic approach focused on the pathophysiology of the disease with a non-aggressive ventilation strategy and conducted by exploiting the advantages of decubitus changes, have led to this exceptional result.
